# GNSS Spoofing Detection via Self-Consistent Verification of Receiver’s Clock State

**DOI:** 10.3390/s26020397

**Published:** 2026-01-08

**Authors:** Yu Chen, Yonghang Jiang, Chenggan Wen, Yan Liu, Linxiong Wang, Xinchen He, Yunxiang Jiang, Xiangyang Peng, Xingqiang Liu, Rong Yang, Jiong Yi

**Affiliations:** 1 College of Semiconductors (College of Integrated Circuits), Changsha Semiconductor Technology and Application Innovation Research Institute, Hunan University, Changsha 410012, China; chenyu1176@hnu.edu.cn (Y.C.); jyh123@hnu.edu.cn (Y.J.); wenchenggan@hnu.edu.cn (C.W.); wlx111@hnu.edu.cn (L.W.); hxc2025@hnu.edu.cn (X.H.); liuxq@hnu.edu.cn (X.L.); 2 Changsha Jinwei Integrated Circuit Co., Ltd., Changsha 410205, China; liuyan@cs-jinwei.com (Y.L.); jiangyunxiang@cs-jinwei.com (Y.J.); pxyfly@126.com (X.P.); 3 College of Computer, National University of Defense Technology, National University of Defense Technology, Changsha 410073, China

**Keywords:** GNSS, spoofing detection, Doppler shift, pseudorange, clock bias, clock drift

## Abstract

Global Navigation Satellite System (GNSS) signals are highly vulnerable to spoofing attacks, which can cause positioning errors and pose serious threats to user receivers. Therefore, the development of efficient and reliable spoofing detection techniques has become an urgent requirement for ensuring GNSS security. In spoofing attacks, attackers introduce additional bias in the Doppler shift. However, detection methods that rely on extracting this deviation from raw measurements suffer from limited practicality, and existing alternative detection schemes based on position, velocity, and time (PVT) information exhibit poor adaptability to diverse scenarios. To address these limitations, this paper proposes a spoofing detection method based on the self-consistency verification of the receiver’s clock state (SCV-RCS). Its core statistic is the cumulative difference between the estimated clock bias and the bias obtained by integrating clock drift. By monitoring this consistency, SCV-RCS identifies anomalies in pseudorange and Doppler observations without complex bias extraction or auxiliary hardware, ensuring easy deployment. Simulation and experimental results demonstrate the method’s effectiveness across diverse spoofing scenarios. It achieves the fastest alarm delay of ≤2 s while providing continuous alerting capability in full-channel and partial-channel spoofing. This study provides a robust and reliable solution for GNSS receivers operating in complex spoofing environments.

## 1. Introduction

With the development and refinement of Global Navigation Satellite Systems (GNSSs), GNSSs can provide massive users with continuous, worldwide coverage of precise position, velocity, and time (PVT) information [1,2,3,4]. GNSSs play a vital role not only in military applications but also in critical civilian fields such as transportation, power grid inspection, emergency response, and smart logistics [5,6,7,8]. However, the extremely low signal strength and open signal structure of GNSS civilian signals make them an ideal entry point for malicious spoofing attacks [9,10,11]. Such attacks aim to manipulate the PVT solutions of target receivers without detection, thereby disrupting normal operations and posing serious threats to various GNSS users [12,13,14]. Consequently, conducting research on accurately detecting GNSS spoofing attacks has become a key issue for ensuring the security of navigation services and promoting the sustainable development of GNSS applications.

Doppler shift reflects the relative velocity between a satellite and a user’s receiver [15,16]. Under normal conditions, due to the high predictability of satellite orbits and the consistency of signal propagation paths, the Doppler shift in GNSS signals aligns with the receiver’s actual motion. In spoofing scenarios, however, it is difficult for attackers to estimate the user’s true state accurately and in real time [17]. As a result, the counterfeit signals they generate are affected by the relative motion between the spoofing device and the user, introducing unavoidable deviations in the observed Doppler shift. Therefore, Doppler bias modeling and analysis have emerged as effective methods to detect spoofing signals [18,19,20,21,22,23,24], particularly in dynamic scenarios.

In the common single-antenna spoofing scenario, the spoofed signal is transmitted from a single antenna and reaches the receiver through a shared propagation path [25]. Based on this setup, some methods attempt to detect spoofing by examining the correlation of Doppler shift across different channels [18,19]. To mitigate interference from satellite motion and receiver clock drift, some studies have attempted to extract implicit Doppler bias from raw measurement using interpolation [20]. However, this method assumes an initialization process under spoofing conditions and a stationary user, which limits its practical applicability. In addition, discrimination is achieved by computing the first-order difference in Doppler shift across channels [21]. In this approach, authentic signals exhibit nonlinear frequency difference due to the receiver motion, while spoofed signals appear linear as Doppler biases cancel out. Nevertheless, this technique requires significant nonlinear motion from the receiver and fails in partial spoofing scenarios where only a channel is affected. To improve detection performance, Doppler ripple has been proposed as a feature, which depends on vertical reciprocating motion of the receiver and may result in false alarms in typical scenarios such as smooth vehicle driving [22]. Although dual-difference methods [23] and multi-receiver architectures [24] have been developed to address these limitations, they often demand complex hardware setups, posing challenges for real-world deployment.

The above studies typically extract Doppler bias directly from raw measurements. As these deviations are deeply embedded in the original observations, additional processing is required for their extraction, which reduces the practical applicability. In fact, Doppler bias not only affect the Doppler measurements themselves but also interfere with the PVT solution [26]. Clock drift, a shared component of Doppler shift, has been proposed as an alternative detection metric by monitoring anomalies in its estimated values [27]. Additionally, the impact of spoofing on navigation integrity can be indirectly assessed by analyzing Doppler positioning residuals [28]. By comparing the Doppler velocity measurement with the rate of change in pseudorange [29], or the results of direct velocity determination and indirect velocity determination [17], inconsistencies introduced by spoofing in pseudorange and Doppler measurements can also be identified. However, these techniques have limited applicability and struggle to effectively address both full-channel and partial-channel spoofing scenarios. Furthermore, their detection performance is highly dependent on the relative velocity between the user and the spoofer. In low-speed or quasi-static environments, the detection probability may drop significantly.

Based on the above considerations, this paper proposes a spoofing detection technique grounded in the self-consistent verification of receiver’s clock state (SCV-RCS). The method detects anomalies introduced by spoofed signals in pseudorange and Doppler observations by monitoring the consistency between clock bias and clock drift. Under normal conditions, these clock parameters are governed by the physical characteristics of the receiver’s local oscillator and follow a stable intrinsic correlation. In this case, both pseudorange and Doppler observations accurately reflect the geometric relationship and relative motion between the user and the satellite, resulting in self-consistent clock parameter solutions. In contrast, under spoofing conditions, spoofed signals inject additional delay into the pseudorange measurement and induce abnormal bias in Doppler shift. This disrupts the inherent consistency between pseudorange and Doppler shift, leading to a breakdown in the correlation between clock bias and drift in the estimation process. Leveraging this characteristic, the proposed method constructs a self-consistency detection metric, SCV-RCS, for clock states to achieve effective spoofing detection. This method inherently bypasses the need for complex bias extraction from raw measurements and requires no auxiliary hardware, resulting in high practical applicability. Extensive evaluations were carried out through simulation, semi-hardware testing, and real-data experiments using the TEXBAT dataset. The results show that this method is applicable to various spoofing attack scenarios and does not rely on the motion state of the receiver. It has technical advantages such as low detection delay, stable performance and continuous detection. This provides a practical and efficient approach for enhancing the anti-spoofing capabilities of GNSS receivers.

The remainder of this paper is structured as follows: Section 2 describes the signal model. Section 3 presents the proposed detection method. Section 4 and Section 5 provide extensive simulation and experimental results along with analysis under various conditions. Finally, Section 6 concludes the paper.

## 2. Signal Model

This section presents a detailed analysis of how spoofed signals systematically corrupt pseudorange and Doppler measurements. To provide analytical clarity and facilitate geometric interpretation, all signal propagation and processing time delays referenced in this work are converted into equivalent spatial distances by multiplication with the speed of light, with results uniformly expressed in meters. To synchronize the analysis with clock bias variations, clock drift is similarly multiplied by the speed of light to obtain an equivalent velocity.

### 2.1. Spoofing Attack Model

**Figure 1:**
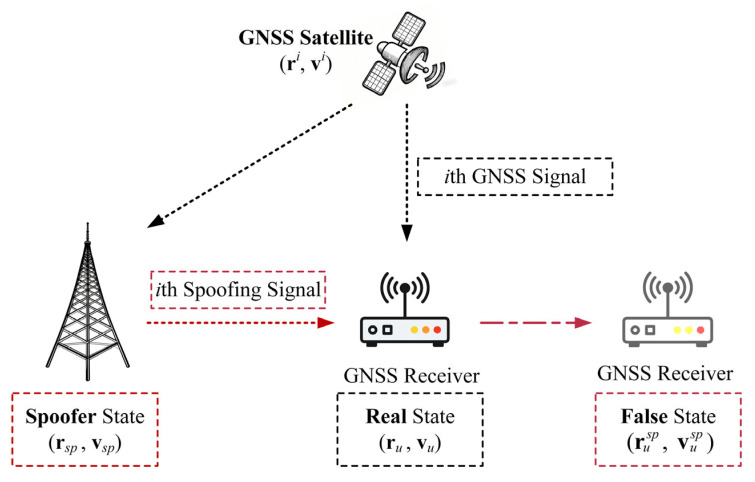
A typical GNSS spoofing scenario.

Figure 1 shows a typical single-antenna GNSS spoofing scenario. In the absence of spoofing, the receiver processes authentic GNSS signals, resulting in a correct PVT solution that reflects the real state. However, under the spoofing attacks, the spoofer processes GNSS signals (e.g., deliberate delay, power amplification) and retransmits the spoofing signals to the target receiver [30]. Since the spoofed signals introduce biased pseudorange and Doppler measurements, and target receiver cannot readily distinguish them from authentic ones, the receiver computes an erroneous PVT solution, thereby entering a manipulated false state.

### 2.2. Doppler Under Spoofing Attack

For the *i*-th real GNSS signal, the Doppler shift $f_{d}^{au,i}$ generated by the relative motion between the satellite and the receiver can be is given by

(1) $$f_{d}^{au,i}={\left(v^{i}-v_{u}\right)}^{T}\cdot \frac{r^{i}-r_{u}}{\left\|r^{i}-r_{u}\right\|}\cdot \frac{f_{c}}{c}$$

where $r^{i}$,$\;v^{i}$, $r_{u}$, and $v_{u}$ represent the satellite position, satellite speed, receiver position and receiver speed, respectively. $f_{c}$ is the carrier frequency and c is the speed of light. The superscript *i* indicates the *i*-th signal.

Considering the influence of clock drift df and noise ${\dot{\varepsilon }}^{i}$, the actual measured Doppler shift becomes

(2) $$f_{d,real}^{au,i}=f_{d}^{au,i}+f_{c}\cdot df+{\dot{\varepsilon }}^{i}=I_{u}^{i}\cdot \frac{f_{c}}{c}+f_{c}\cdot df+{\dot{\varepsilon }}^{i}$$

The Doppler frequency shift corresponding to the false state can be expressed as

(3) $$f_{d}^{sp,i}={\left(v^{sp,i}-v_{u}^{sp}\right)}^{T}\cdot \frac{r^{sp,i}-r_{u}^{sp}}{\left\|r^{sp,i}-r_{u}^{sp}\right\|}\cdot \frac{f_{c}}{c}=I_{u}^{sp,i}\cdot \frac{f_{c}}{c}$$

where $r_{u}^{sp}$ and $v_{u}^{sp}$, respectively, denote the position and velocity of the receiver under false state. $r^{sp,i}$ and $v^{sp,i}$, respectively, denote the position and velocity of the spoofed satellite by spoofer. $I_{u}^{sp,i}$ is defined as the relative velocity of the *i*th spoofed satellite and the false state.

The transmitted carrier frequency of the spoofing signal is subsequently adjusted according to $f_{d}^{sp,i}$; that is,

(4) $$f^{sp,i}=f_{c}-f_{d}^{sp,i}$$

Taking into the relative motion between the spoofer and the receiver, the relationship between the resulting Doppler shift $f_{sp,u}^{i}$ and broadcast frequency can be formulated as

(5) $$f_{sp,u}^{i}={\left(v_{sp}-v_{u}\right)}^{T}\cdot \frac{r_{sp}-r_{u}}{\left\|r_{sp}-r_{u}\right\|}\cdot \frac{f^{sp,i}}{c}=I_{sp,u}\cdot \frac{f_{c}-I_{u}^{sp,i}\cdot \frac{f_{c}}{c}}{c}\approx I_{sp,u}\cdot \frac{f_{c}}{c}$$

where $r_{sp}$ and $v_{sp}$, respectively, denote the position and velocity of the spoofer. $I_{sp,u}$ is defined as the relative velocity of the spoofer and receiver.

In summary, the actual Doppler shift $f_{d,real}^{sp,i}$ measured by the receiver under spoofing attacks can be expressed as

(6) $$f_{d,real}^{sp,i}=f_{c}-\left(f^{sp,i}-f_{sp,u}^{i}\right)+f_{c}\cdot df+{\dot{\varepsilon }}^{i}=f_{d}^{sp,i}+f_{c}\cdot df+f_{sp,u}^{i}+{\dot{\varepsilon }}^{i}\;=f_{d}^{sp,i}+f_{c}\cdot df+I_{sp,u}\cdot \frac{f_{c}}{c}+{\dot{\varepsilon }}^{i}=f_{d}^{sp,i}+f_{c}\cdot df+\Delta f_{d,i}$$

The Doppler measurement in Equation (6) comprises three distinct components: the Doppler shift $f_{d}^{sp,i}$ induced by the false state, the contribution from receiver clock drift $f_{c}\cdot df$, and the additional Doppler bias $\Delta f_{d,i}$ introduced by the spoofing signal, which is defined as

(7) $$\Delta f_{d,i}=I_{sp,u}\cdot \frac{f_{c}}{c}+{\dot{\varepsilon }}^{i}$$

This Doppler bias originates from the relative motion between the spoofer and the receiver. The magnitude of this bias is directly proportional to the relative speed between them.

### 2.3. Pseudorange Under Spoofing Attack

Similarly, the reception-and-retransmission mechanism inherent to the spoofer inevitably introduces additional bias into the pseudorange measurement.

For the *i*-th authentic GNSS signal, its pseudorange can be expressed as follows [31]:

(8) $$p^{au,i}=\|r^{i}-r_{u}\|+dt-dt^{i}+I^{i}+T^{i}+\varepsilon^{i}$$

where dt, $dt^{i}$, $I^{i}$, $T^{i}$, and $\varepsilon^{i}$ represent the clock bias, satellite drift, ionospheric delay, tropospheric delay and measurement noise, respectively.

Under spoofing conditions, the pseudorange of the *i*-th spoofing signal is given by

(9) $$p^{sp,i}=\|r^{i}-r_{sp}\|+\|r_{sp}-r_{u}\|+dt+\Delta t^{sp,i}-dt^{i}+I^{i}+T^{i}+\varepsilon^{i}$$

where $\Delta t^{sp,i}$ is the additional processing delay intentionally introduced by the spoofer to mislead the receiver.

Equation (9) can be reformulated to explicitly reveal the introduced pseudorange bias:

(10) $$p^{sp,i}=\|r^{i}-r_{u}\|+dt-dt^{i}+I^{i}+T^{i}+\Delta p^{i}+\varepsilon^{i}=p^{au,i}+\Delta p^{i}$$

where the pseudorange bias $\Delta p^{i}$ is defined as

(11) $$\Delta p^{i}=\|r^{i}-r_{sp}\|+\|r_{sp}-r_{u}\|-\|r^{i}-r_{u}\|+\Delta t^{sp,i}$$

This bias stems from the geometric path difference introduced by the spoofer position and the additional processing delay. By systematically injecting bias into the pseudorange measurement for each satellite, the spoofing signals can cause deviation in the receiver’s PVT solution, thereby achieving the objective of manipulating the receiver into outputting a false state.

## 3. Spoofing Detection Methodology

Based on the analysis of spoofing attack impacts on pseudorange and Doppler observables presented in Section 2, this section formalizes the underlying mechanism by which spoofing disrupts the inherent consistency between receiver’s clock bias and clock drift. Leveraging this theoretical foundation, a spoofing detection scheme grounded in the self-consistency verification of the receiver’s clock state is proposed. After discussing the detection principle in this section, a specific detection statistic, SCV-RCS, is constructed and its detection threshold and theoretical performance are analyzed.

### 3.1. Influence of Consistency Between Receiver’s Clock Bias and Clock Drift

As established in the preceding analysis, spoofing introduces systematic biases into both Doppler and pseudorange measurements. These biases are inherently embedded within the raw observables, posing a significant challenge to conventional direct monitoring techniques. In fact, the impact of these biases is directly manifested in the receiver’s PVT solution, leading to the inconsistency between the estimated clock bias and clock drift.

From Equations (2) and (8), taking into account pseudorange correction, under normal conditions, we have

(12) $$f_{d,real}^{au,i}=f_{d}^{au,i}+f_{c}\cdot df+{\dot{\varepsilon }}^{i}\;p^{au,i}=\|r^{i}-r_{u}\|+dt+\varepsilon_{i}$$

The pseudorange and Doppler shift measurements both reflect the receiver’s real kinematic state, leading to a consistent PVT solution. Therefore, a fundamental self-consistency is established between the estimated clock bias *dt* and clock drift *df*.

From Equations (6) and (9), under spoofing attacks, we have

(13) $$f_{d,real}^{sp,i}=f_{d}^{sp,i}+f_{c}\cdot df+f_{sp,u}^{i}+{\dot{\varepsilon }}^{i}\;p^{sp,i}=\|r^{i}-r_{sp}\|+\|r_{sp}-r_{u}\|+dt+\Delta t^{sp,i}+\varepsilon^{i}$$

where the introduced Doppler bias $f_{sp,u}^{i}$, geometric offset, and the spoofer’s processing delay $\Delta t^{sp,i}$ disrupt the intrinsic geometric model. This corruption breaks the self-consistency relationship between *dt* and *df* that holds under authentic conditions, providing a direct theoretical basis for detecting the spoofing-induced anomaly. The explicit form of this relationship between *dt* and *df* will be derived below.

Assume $f_{d}^{sp,i}$ corresponds to $\left\|r^{i}-r_{sp}\right\|$, eflecting the spoofer’s reception of the real GNSS signal) and the term dt corresponds to *df*, consistently reflects the receiver’s clock state. The fundamental issue then lies with the remaining terms: the geometric range $\left\|r_{sp}-r_{u}\right\|$, the processing delay $\Delta t^{sp,i}$, and the induced Doppler bias $f_{sp,u}^{i}$ exhibit an inherent mismatch. Even if the additional processing delay $\Delta t^{sp,i}$ is disregarded, the relationship between the geometric distance $\left\|r_{sp}-r_{u}\right\|$ and the corresponding Doppler shift $f_{sp,u}^{i}$ is not self-consistent in the receiver’s estimation model.

Specifically, after spoofing attacks occur, as the receiver moves, the geometric range changes from $\left\|r_{sp}-r_{u}\right\|$ to $\|r_{sp}-r_{u}\|+\Delta r$, where Δr is the distance traveled by the receiver and the resulting Doppler bias $f_{sp,u}^{i}$ can be regarded as consistent with Δr. The inherent geometric distance $\left\|r_{sp}-r_{u}\right\|$ between the spoofer and the receiver constitutes the ineliminable inconsistency between the pseudorange and the Doppler measurements, which is the fundamental cause. As long as the spoofer and receiver are not co-located, this bias persists.

In a full-channel spoofing scenario, the common delay $\Delta t^{sp,i}$ and geometric range $\|r_{sp}-r_{u}\|+\Delta r$ between the spoofer and the receiver is superimposed onto the pseudorange of each satellite. This common-mode offset is absorbed by the estimator as an equivalent clock bias, causing the estimated bias to shift from its authentic value dt to a spoofed value $\|r_{sp}-r_{u}\|+\Delta r+dt+\Delta t^{sp,i}$. Concurrently, the Doppler shift $f_{sp,u}^{i}$ induced by the relative motion between the spoofer and the receiver is also added to each satellite’s measurement. This common Doppler component is likewise absorbed as an equivalent clock drift, shifting the estimated drift from df to $df+f_{sp,u}^{i}$/$f_{c}$. This inconsistency will be fully reflected in the clock bias and clock drift.

In a partial-channel spoofing scenario, where some signals are from spoofed satellites and others from authentic ones, the PVT solution minimizes the sum of squared residuals. Consequently, the result does not correspond to either the real or false state but instead converges to a compromised solution. This occurs because the contradiction between the true and spoofed signals cannot be resolved, forcing the estimator to compromise in the PVT solution. As a result, the estimated clock bias and clock drift deviate from their physical relationship. Furthermore, since the Doppler measurement is sensitive to the rate of change in delay rather than to a fixed delay, only the pseudorange is affected, not the Doppler shift. This further exacerbates the inconsistency between clock bias and drift.

### 3.2. Proposed Spoofing Detection Methodology SCV-RCS

**Figure 2:**
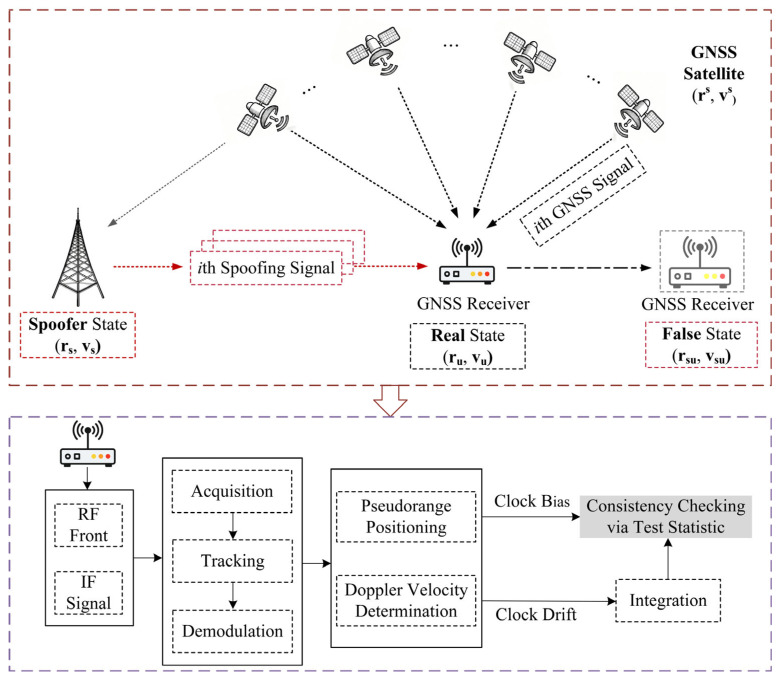
Flowchart of the proposed detection method SCV-RCS.

Building upon the theoretical analysis in Section 3.1, we propose a spoofing detection metric based on the self-consistency between the estimated receiver clock bias and the clock drift, as shown in Figure 2. Under normal conditions, both pseudorange and Doppler measurements originate from consistent satellite–receiver geometry and are jointly used for PVT solutions.

The corrected pseudorange equation system is as follows:

(14) $$\left\{ \begin{array}{c} p^{1}=\|r^{1}-r_{u}\|+dt+\varepsilon^{1}\\ \vdots \\ p^{i}=\|r^{i}-r_{u}\|+dt+\varepsilon^{i}\\ \vdots \\ p^{n}=\|r^{n}-r_{u}\|+dt+\varepsilon^{n} \end{array}\right.$$

This is expressed in a matrix as

(15) F=G·dX

where the residual vector F, geometric matrix G and parameter correction amount dX are defined as

(16) $$F=\left[\begin{array}{c} p^{1}-\|r^{1}-r_{u}\|-dt\\ \vdots \\ p^{i}-\|r^{i}-r_{u}\|-dt\\ \vdots \\ p^{n}-\|r^{n}-r_{u}\|-dt \end{array}\right]$$

(17) $$G=\left[\begin{array}{cccc} \frac{\partial p^{1}}{\partial x_{u}} & \frac{\partial p^{1}}{\partial y_{u}} & \frac{\partial p^{1}}{\partial z_{u}} & 1\\ \vdots & \vdots & \vdots & \vdots \\ \frac{\partial p^{i}}{\partial x_{u}} & \frac{\partial p^{i}}{\partial y_{u}} & \frac{\partial p^{i}}{\partial z_{u}} & 1\\ \vdots & \vdots & \vdots & \vdots \\ \frac{\partial p^{n}}{\partial x_{u}} & \frac{\partial p^{n}}{\partial y_{u}} & \frac{\partial p^{n}}{\partial z_{u}} & 1 \end{array}\right]$$

(18) $$dX=X-X_{0}=\left[\begin{array}{c} x_{u}-x_{u}\left[0\right]\\ y_{u}-y_{u}\left[0\right]\\ z_{u}-z_{u}\left[0\right]\\ dt-dt\left[0\right] \end{array}\right]$$

where $X_{0}={\left[x_{u}\left[0\right],\;y_{u}\left[0\right],\;z_{u}\left[0],\;dt[0\right]\right]}^{T}$ is the state vector of the initial point.

By applying the least-squares method to solve the matrix Equation (15), we can obtain

(19) $$dX={\left(G^{T}G\right)}^{-1}G^{T}F$$

The final estimated receiver clock bias is

(20) $$dt=X_{0}\left(4\right)+dX\left(4\right)$$

where $X_{0}$(4) and dX(4) denote the fourth element of the state vector $X_{0}$ and the correction vector dX, respectively, both corresponding to the clock bias component.

Similarly, the clock drift *df* can likewise be obtained by solving the Doppler observation equation system via the least-squares method. The system, analogous to Equation (14) but formulated for Doppler measurements $f_{d}^{i}$, uses the same geometric matrix G in Equation (15). It can be expressed in matrix form as $\dot{F}=G\cdot dV$, where $dV={\left[\Delta v_{xu},\Delta v_{yu},\Delta v_{zu},\Delta df\right]}^{T}$ contains the velocity and clock drift updates and $\dot{F}$ is the Doppler residual vector. Its least-squares solution is given by $dV={\left(G^{T}G\right)}^{-1}G^{T}\dot{F}$. The final estimated receiver clock drift df is $df=V_{0}\left(4\right)+dV\left(4\right)$, where $V_{0}={\left[V_{xu}\left[0\right],\;V_{yu}\left[0\right],\;V_{zu}\left[0],\;df[0\right]\right]}^{T}$ is the velocity state vector of the initial point.

As a result, the estimated clock bias *dt* and the clock drift *df* reflect the physical characteristics of the receiver’s local oscillator and follow a stable intrinsic correlation, resulting in self-consistent clock bias and the clock drift. However, in the presence of spoofing attacks, due to the geometric distance between the spoofer and the receiver, the injected bias in pseudorange and the Doppler shift break this consistency.

To detect this inconsistency, we propose a detection statistic based on the difference between the directly estimated clock bias *dt* and the clock bias obtained by integrating the estimated clock drift *df* over time. The statistic in the time epoch *k* is expressed as

(21) $$\delta \left[k\right]=dt\left[k\right]-dt^{int}\left[k\right]$$

where $dt^{int}\left[k\right]$ is the integrated clock bias by clock drift *df*, indicated as

(22) $$dt^{int}\left[k\right]=dt\left[0\right]+\overset{k}{\underset{j=1}{\sum }}df\left[k\right]\cdot \Delta t$$

where Δt is the epoch interval, with a typical range of 0.05 s to 1 s. In the subsequent study of this paper, the value Δt = 0.1 s is selected to balance dynamic adaptability of commercial receivers, fulfillment of real-time spoofing detection requirements, and comparability with existing studies.

Therefore, the spoofing detection problem can be modeled as a hypothesis test:

(23) $$\left\{ \begin{array}{cc} H_{0}:\delta \left[k\right]∼N\left(0,\sigma_{0}^{2}\right), & Spoofing-free\\ H_{1}:\delta \left[k\right]∼N\left(s_{t},\sigma_{1}^{2}\right), & Spoofing \end{array}\right.$$

where $H_{0}$ is the spoofing-free condition, and $H_{1}$ represents the spoofing condition. $\sigma_{0}^{2}$ and $\sigma_{1}^{2}$ denote the combined influence of Doppler and pseudorange measurement noise under the different conditions. $s_{t}$ is an inconsistent bias introduced by spoofing attacks, which is related to the distance $\left\|r_{sp}-r_{u}\right\|$ between the spoofer and the receiver, the additional processing delay $\Delta t^{sp,i}$, and the state of the spoofing channel configuration (partial or full). Their estimated values can be modeled as

(24) $$\left\{ \begin{array}{l} {\hat{\sigma }}_{0}^{2}=\frac{1}{K}\overset{K-1}{\underset{k=0}{\sum }}\delta {\left[k\right]}^{2}\\ {\hat{\sigma }}_{1}^{2}=\frac{1}{K}\overset{K-1}{\underset{k=0}{\sum }}{\left(\delta \left[k\right]-\hat{s_{t}}\right)}^{2}\\ {\hat{s}}_{t}=\frac{1}{K}\overset{K-1}{\underset{k=0}{\sum }}\delta \left[k\right] \end{array}\right.$$

Under spoofing-free conditions, the receiver’s *dt* and *df* is governed by a stable oscillator, and the two estimates should be consistent. $\delta \left[k\right]$ is only affected by measurement noise. However, under spoofing conditions, the injected geometric and kinematic biases cause a systematic deviation $s_{t}$ between the two clock bias estimates.

To reduce false alarms, we accumulate instantaneous inconsistencies in a sliding window of V epochs to construct a cumulative test statistic:

(25) $$T\left[k\right]=\overset{V-1}{\underset{l=0}{\sum }}\delta {\left[k-l\right]}^{2}$$

The probability distribution of *T* satisfies

(26) $$\left\{ \begin{array}{cc} H_{0}:\frac{T}{\sigma_{0}^{2}}∼\chi_{L}^{2}, & Spoofing-free\\ H_{1}:\frac{T}{\sigma_{1}^{2}}∼\chi_{L}^{\prime 2}\left(\lambda \right), & Spoofing \end{array}\right.$$

where $\chi_{L}^{2}$ and $\chi_{L}^{\prime 2}$ are the central and noncentral chi-squared distribution with V degrees of freedom, respectively. λ is the noncentral parameter, defined as

(27) $$\lambda =\overset{V-1}{\underset{l=0}{\sum }}s_{t}{\left(k\right)}^{2}$$

### 3.3. Detection Threshold and Detection Performance

By presetting the false alarm probability (Pfa), the detection threshold γ is obtained as follows:

(28) $$\gamma =\sigma_{0}^{2}\cdot Q_{\chi_{L}^{2}}^{-1}\left(\mathrm{Pfa}\right)$$

where $Q_{\chi_{L}^{2}}^{-1}$ is the inverse function of the right-tail probability of the chi-square distribution.

Subsequently, the corresponding detection probability can be derived:

(29) $$P_{D}=Q_{\chi_{L}^{\prime 2}}\left(\frac{\gamma }{\sigma_{1}^{2}}\right)$$

where $Q_{\chi_{L}^{\prime 2}}$ is the right-tail probability of the noncentral chi-square distribution.

**Figure 3:**
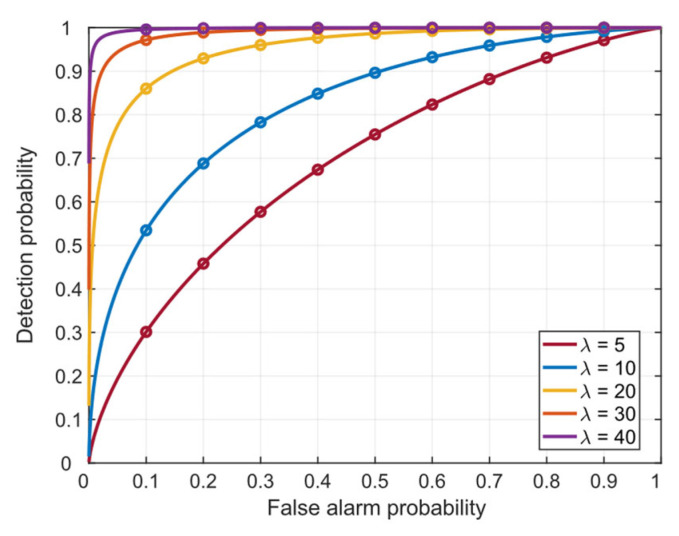
ROC curves with different values of λ.

Figure 3 presents the theoretical receiver operating characteristic (ROC) curves of the detector under various values of λ. It can be observed that, for a fixed false alarm probability Pfa, a larger non-centrality parameter λ results in a higher detection probability, making it easier to detect spoofed signals.

## 4. Simulation Verification

This section describes how simulation-based verification of the proposed algorithm was conducted under numerous diverse spoofing scenarios, including partial-channel spoofing, full-channel spoofing, and time synchronization attack (TSA) scenarios. TSA is a special deception scenario that affects the clock difference rather than the position by introducing a common delay on the pseudorange of each satellite. Throughout the process, authentic GNSS signals were simulated and combined with spoofing signals featuring various replaying delays and channel configurations before being sampled. The resultant IF signals were subsequently processed using the FGI-GSRx-2.0.1 software receiver [32], with codes downloaded from https://github.com/nlsfi/FGI-GSRx (accessed on 15 July 2025).

### 4.1. Partial-Channel Spoofing Simulation

**Figure 4:**
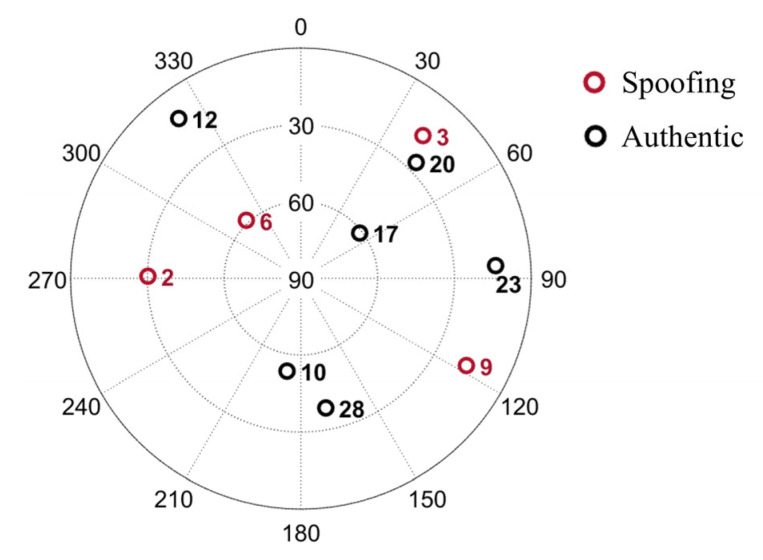
Skyplot for the used GPS satellites.

This section details 12 distinct scenarios (case 1 to case 12), defined by combinations of receiver status and spoofing signal replaying delay. The spoofing attack begins at 14 s, when the spoofer is positioned 100 m from receiver. Key configuration parameters are detailed in Table 1, and Figure 4 illustrates the satellite skyplot at the simulation’s commencement.

**Figure 5:**
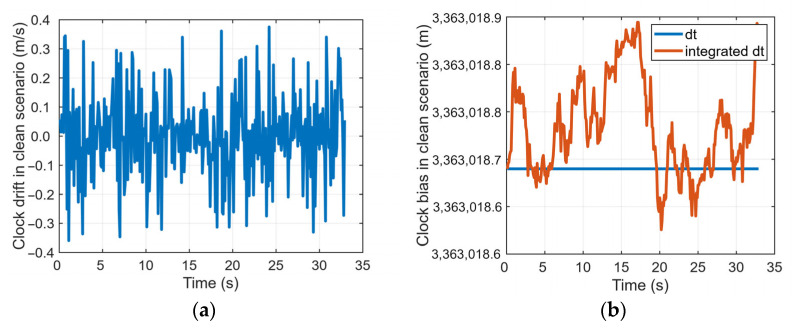

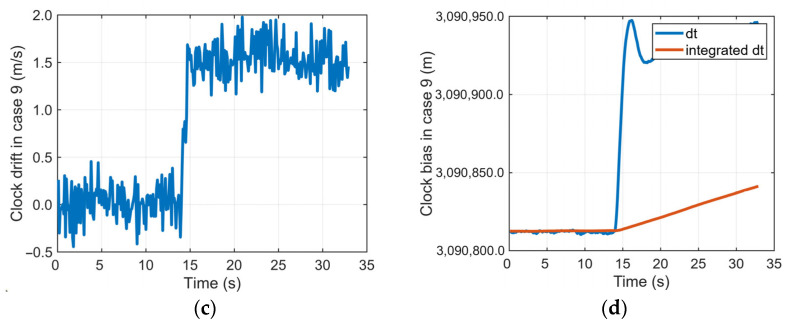
Clock states in different scenarios: (**a**) clock drift in clean scenario; (**b**) clock bias and integrated clock bias in clean scenario; (**c**) clock drift in case 9; (**d**) clock bias and integrated clock bias in case 9.

Figure 5 uses the clean scenario and case 9 as examples to compare the relationship between the receiver clock bias *dt* and *dt* integrated by the clock drift *df*. The results indicate that in the clean scenario, the two clock bias values are highly consistent. In the spoofing scenario, however, this consistency is significantly disrupted after the attack begins, exhibiting a clear deviation. This deviation characteristic can serve as an effective feature for identifying GNSS spoofing.

**Figure 6:**
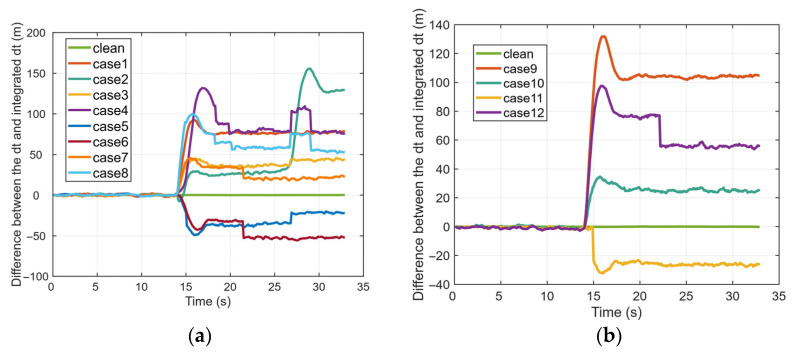
Difference between the dt and integrated dt in different scenarios: (**a**) case 1 to case 8; (**b**) case 9 to case 12.

Figure 6 shows the difference between *dt* and integrated *dt* in the spoofing scenarios of case 1 to case 12. Prior to the onset of spoofing, their difference approximates zero, which indicates normal system operation. Following the initiation of the spoofing attack, however, a significant and immediate deviation emerges between dt and integrated dt, with the magnitude of change varying across the different scenarios.

**Figure 7:**
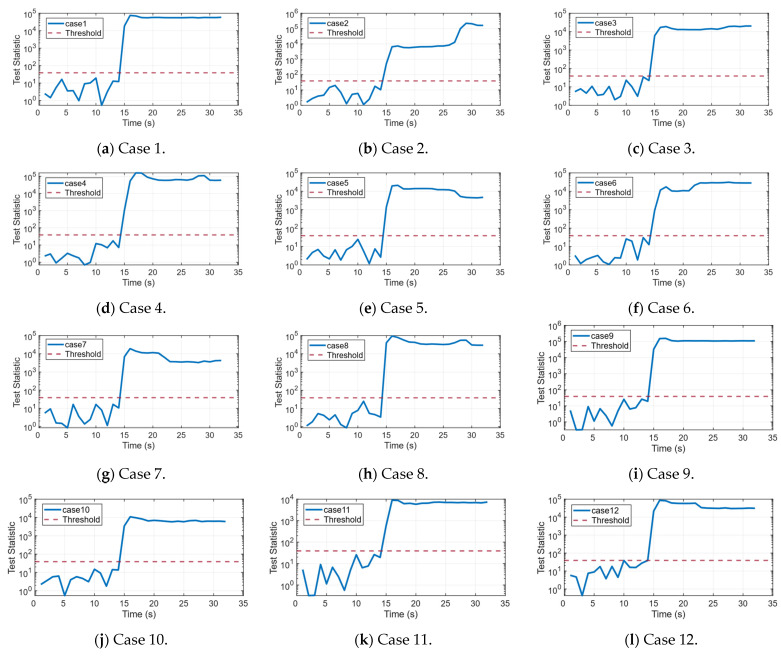
Test statistic SCV-RCS of different scenarios from case 1 to case 12.

To further evaluate the spoofing detection performance of the proposed method, Figure 7 presents the temporal variation in the test statistic SCV-RCS across case 1 to case 12. Prior to the spoofing attack (i.e., before 14 s), the statistic in every scenario remains below the predefined threshold (red dashed line), indicating normal system operation. Following the attack’s initiation, the statistic in all scenarios rises rapidly, significantly exceeding the threshold within 2 s. The resulting abrupt change enables the detector to accurately identify an attack shortly after its onset, providing timely warning. This demonstrates a high sensitivity and a pronounced response to spoofing.

The simulation results confirm that the proposed detection method exhibits favorable response characteristics under various configurations in the partial-channel spoofing scenarios. It is capable of rapidly issuing an assessment after an attack begins, demonstrating both good real-time performance and accuracy.

### 4.2. Full-Channel Spoofing Simulation

This section presents the simulation verification of full-channel spoofing scenarios, including 8 different scenarios (cases 1 to 8), with all spoofing signals in each scenario having a replaying delay added. Table 2 details the configuration parameters that differ from those in Table 1.

**Figure 8:**
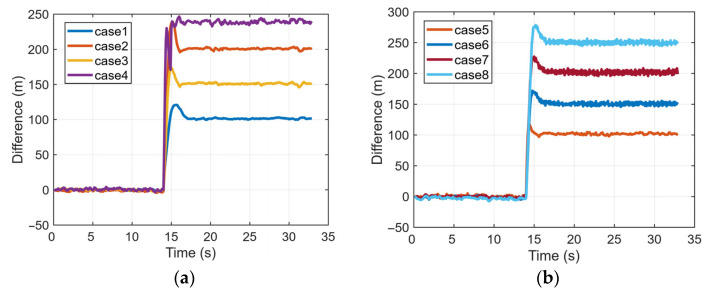
Difference between the dt and integrated dt in different scenarios: (**a**) case 1 to case 4; (**b**) case 5 to case 8.

Figure 8 shows the difference between *dt* and integrated *dt* in the spoofing scenarios of case 1 to case 8. In all scenarios except cases 3 and case 4, each satellite is subjected to an identical added delay. This delay, combined with the geometric range between the spoofer position and the receiver, is jointly absorbed into the clock bias *dt* without affecting the clock drift *df*. The resulting differences align with the theoretical analysis presented in Section 2, exhibiting the expected stepwise increase that corresponds to the incrementally applied delays.

Simulation results for the full-channel spoofing scenario confirm that the detection performance of the proposed method is consistent with that observed in partial-channel scenarios. The method rapidly detects an attack upon its occurrence, with an alarm time of less than 2 s, demonstrating that both its real-time performance and accuracy meet application requirements.

### 4.3. TSA Simulation

**Figure 9:**
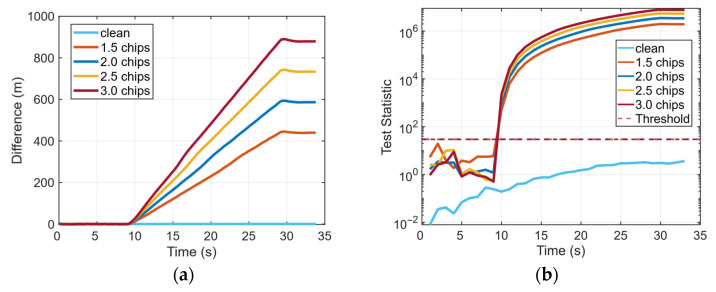
Difference between the dt and integrated dt and test statistic with different time pushes under TSA scenarios: (**a**) Difference between the dt and integrated dt. (**b**) Test statistic, SCV-RCS.

Figure 9 presents the difference between *dt* and integrated *dt* for TSA scenarios under different time pushes with 1.5 to 3.0 chips, along with the corresponding temporal variation in the detection statistic.

The time pull begins in the 9th s; the difference in Figure 9a rapidly diverges from the clean reference, and the difference exhibits sustained accumulation and shows a positive correlation with time. This is because when designing the TSA scenario, the spoofing rate was configured to have a linear relationship with time. Concurrently, the detection statistic SCV-RCS in Figure 9b rises rapidly after the attack begins, exceeding the predefined threshold in all cases and thus successfully triggering a spoofing detection. These results indicate that the proposed method possesses favorable sensitivity and detection performance against TSAs.

## 5. Experimental Results and Analysis

To further validate the effectiveness and applicability of the proposed spoofing detection algorithm in practical scenarios, this section presents both semi-hardware simulation and real-world data testing. These experiments were designed to evaluate the performance of the method under hardware-in-the-loop conditions and authentic spoofing environments. Meanwhile, through comparative analysis with existing mainstream algorithms, the superiority of the proposed method is highlighted.

### 5.1. Semi-Hardware Simulation

**Figure 10:**
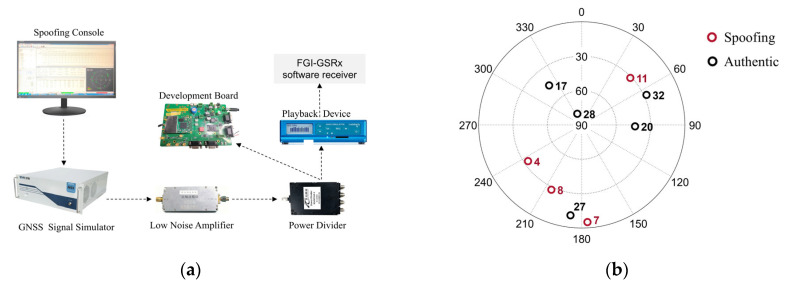
Experimental platform environment. (**a**) Experimental platform. (**b**) Skyplot for the used GPS satellites.

A semi-hardware experimental platform was built to emulate realistic spoofing conditions with high controllability. As shown in Figure 10a, the platform consists of a GNSS signal simulator, a spoofing console, RF front-end hardware components (including a low-noise amplifier and power divider), a development board, and a playback device. The spoofing signal is generated in real time by the control console, replayed with configurable parameters, and combined with authentic GNSS signals for testing. The control console serves as the core of the signal generation system, providing a user interface for system interaction. It controls the generation of both authentic and spoofing signals, including spoofing satellite numbers, location, and power settings. The simulator generates authentic GNSS signals and spoofing signals the preset ephemeris, respectively, and combines the two signals. The combined signal is output the navigation signal recording and playback device for high-fidelity data sampling and storage. The IF signal stored in the navigation signal recording and playback equipment will be transmitted to the software receiver for processing and spoofing detection. The function of the development board is to verify if the spoofing signal complies with the specified requirements.

To simulate a realistic partial-channel spoofing environment (Group 1), several satellites were designated as spoofed or authentic, as illustrated in Figure 10b. Group 2 is a full-channel spoofing scenario, meaning that all signals received after the spoofing attack begins are spoofing signals. Other key simulation parameters are summarized in Table 3.

**Figure 11:**
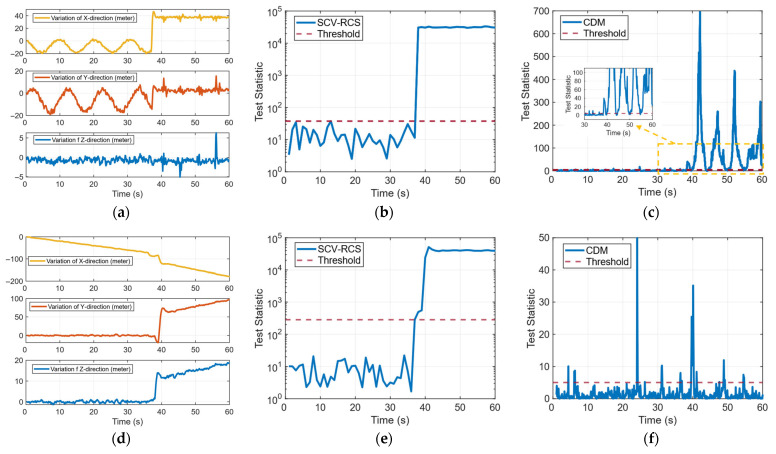
Comparison of the test results of case 1 and case 5. (**a**) Positioning change of case 1. (**b**) SCV-RCS statistic of case 1. (**c**) CDM statistic of case 1. (**d**) Positioning change of case 5. (**e**) SCV-RCS statistic of case 5. (**f**) CDM statistic of case 5.

Take case 1 and case 5 in the two groups as examples to compare the test results. Case 1 represents a uniform circular motion, while case 5 corresponds to a uniform linear motion along the x-axis. At the onset of spoofing, the receiver is positioned 50 m from the spoofer position. Subsequently, under the full-channel spoofing configuration, the receiver’s position solution rapidly converges on the location of the spoofer position, as shown in Figure 11a,d.

Figure 11c,f present the temporal variation in the detection statistic based on the clock drift monitoring (CDM) technique [27]. The results indicate that in both scenarios, the CDM statistic responds to the spoofing attack, with the statistic exceeding the predefined threshold, thereby demonstrating a capability for spoofing detection. However, the statistical response in case 1 is more pronounced and sustained. Multiple distinct peaks occur after the attack, indicating good detectability, though instances of missed detection are also present. For case 5, while the statistic exceeds the threshold at certain points, its overall fluctuation is minor and its amplitude limited. Consequently, some attack segments fail to trigger an effective detection. These results demonstrate that the CDM metric is highly dependent on receiver motion state and the spoofing scenario, with case 5 exhibiting a significantly higher risk of both missed and false detection compared with case 1.

The performance of the SCV-RCS statistic for the same two cases is shown in Figure 11b,e. The SCV-RCS statistic remains below the threshold before the attack, confirming robustness under normal conditions. Upon spoofing activation, it rises sharply and stably exceeds the threshold in both scenarios. In full-channel spoofing (case 1), the statistic increases by several orders of magnitude and maintains a consistently high level. Even in the low-dynamic and partial-channel spoofing (case 5), the SCV-RCS statistic still exhibits a rapid and stable rise, reliably identifying the spoofing event.

These results demonstrate that the proposed SCV-RCS method delivers stable, accurate detection across different channel and motion patterns, significantly outperforming the conventional CDM technique in reliability and robustness.

**Figure 12:**
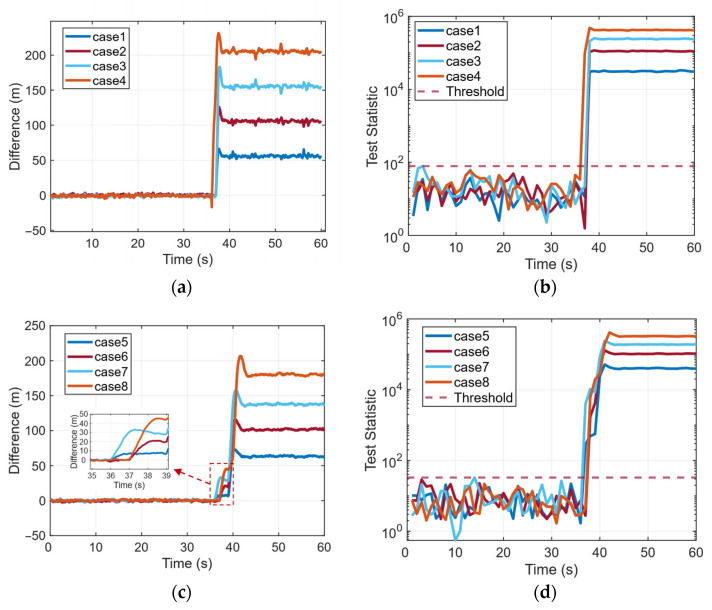
Difference between *dt* and integrated *dt* and SCV-RCS statistic under different scenarios: (**a**) Difference between dt and integrated dt of case 1 to case 4. (**b**) SCV-RCS statistic of case 1 to case 4. (**c**) Difference between dt and integrated dt of case 5 to case 8. (**d**) SCV-RCS statistic of case 5 to case 8.

Figure 12 further evaluates the performance of the proposed SCV-RCS method across all spoofing scenarios from case 1 to case 8. As shown in Figure 12a,c, the difference between the raw clock bias and the integrated clock drift remains close to zero before the spoofing attack, indicating a high degree of internal consistency under normal conditions. After the attack begins (around 37 s), all cases exhibit a rapid and stable divergence in this difference, serving as a strong indicator of spoofing-induced inconsistencies.

Correspondingly, Figure 12b,d present the SCV-RCS detection statistic over time. Across all scenarios, the statistic consistently remains below the detection threshold prior to the attack and rapidly rises above the threshold immediately after spoofing is initiated. The test statistic not only surpasses the threshold within 2 s in every case but also stabilizes at a high level, clearly distinguishing spoofed conditions from normal ones.

These results demonstrates that the proposed SCV-RCS approach maintains robust spoofing detection capability across a variety of receiver motion patterns and spoofing configurations, including circular and linear motion, partial-channel and full-channel spoofing, and various replaying delays.

### 5.2. TEXBAT Test

To further evaluate the performance of the proposed method under real-world TSA condition, the publicly available TEXBAT dataset is utilized [33]. Specifically, the ds3 scenario is selected, which represents a challenging spoofing case with gradual takeover. Four typical spoofing detection statistics are compared: Q-energy [34,35], MSCD [36], VSQM [37], and the proposed SCV-RCS.

**Figure 13:**
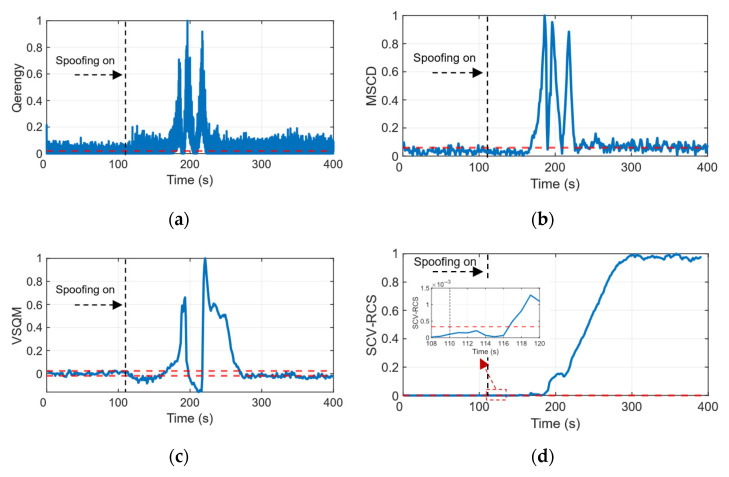
Comparison of the test statistics under ds3 scenario (red dashed line is detection threshold). (**a**) Q-energy statistic. (**b**) MSCD statistic. (**c**) VSQM statistic. (**d**) SCV-RCS statistic.

To quantitatively compare the detection speed and stability, we examine both the first stable alarm and the detectable time segment. As shown in Figure 13, all evaluated metrics respond to the spoofing events to some degree. The MSCD (b) and VSQM (c) statistics show first stable alarms with delays of approximately 58 s and 10 s after the spoofing onset (~110 s), respectively. The Q-energy statistic (a) achieves a faster initial alarm (delay of ~1 s). However, its detection is not sustained, as the statistic frequently falls below the threshold thereafter, indicating high instability and a significant risk of missed detection. In fact, these three benchmark metrics exhibit distinct peaks during the 150–250 s interval, but they also exhibit a certain period of missed detection (i.e., after 250 s). They fail to reach the detection threshold during several attack segments. In contrast, the proposed SCV-RCS statistic (d) remains stable before the attack, rises rapidly and steadily after the spoofing begins, and first crosses the detection threshold stably at around 117 s, resulting in a consistent alarm delay of approximately 7 s. More importantly, once stably triggered, the SCV-RCS statistic maintains its alarm state with far greater persistence than the benchmark methods. While minor fluctuations are observed within the first ~60 s, the statistic quickly stabilizes and then maintains sustained, continuous exceedance above the threshold for the remainder of the spoofing duration, achieving near-complete coverage of the attack period with no missed detection. This comparative analysis clearly indicates the superior robustness and sensitivity of SCV-RCS, which offers not only a competitively fast response but also a drastically reduced risk of missed detection compared to the existing methods.

**Figure 14:**
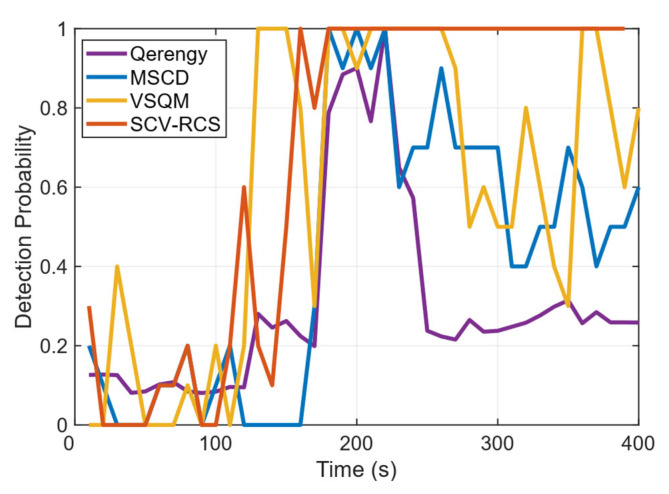
Comparison of the detection probability under ds3 scenario.

To further quantify the detection performance, Figure 14 compares the temporal evolution of the detection probability for each method. The proposed SCV-RCS statistic achieves a detection probability of 60% within 10 s of the spoofing onset (110 s) and maintains a level above 80% after 50 s, approaching 100% within 60 s and sustaining it thereafter. In comparison, the VSQM statistic demonstrates a competitive response, reaching approximately 70% probability shortly after the attack begins. However, its performance degrades significantly after 50 s, exhibiting instability in the latter phase. Similarly, the MSCD statistic shows considerable fluctuation throughout the attack duration. The Q-energy statistic, in contrast, exhibits generally poor sensitivity with the lowest detection probability among all methods. Overall, SCV-RCS provides the most stable, reliable, and sustained high detection probability across the entire spoofing interval.

**Figure 15:**
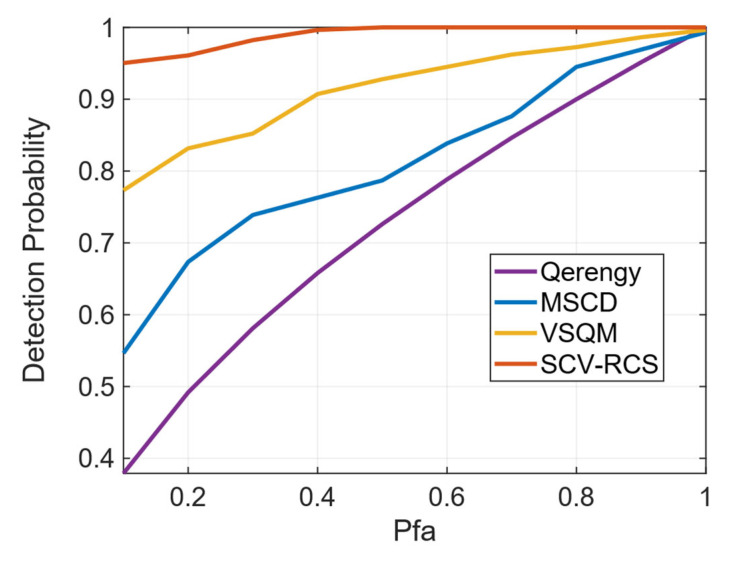
Comparison of ROC curves under ds3 scenario.

The ROC curves in Figure 15 provide a comprehensive quantitative comparison of the detection performance, illustrating the fundamental trade-off between detection probability and false alarm rate for all four methods. As shown in Figure 15, the ROC curve of the proposed SCV-RCS method consistently dominates those of the benchmark methods across the entire range of Pfa. This signifies that, for any given acceptable level of false alarms, SCV-RCS achieves a significantly higher probability of correctly detecting the spoofing attack. A concrete example illustrates this advantage: at a Pfa of 10%, SCV-RCS attains a detection probability of 95.03%, vastly outperforming VSQM (77.31%), MSCD (54.63%), and Q-energy (37.09%). This ROC analysis provides consolidated quantitative evidence that SCV-RCS offers a superior detection capability.

These results demonstrate that the proposed SCV-RCS method significantly outperforms conventional techniques under realistic spoofing conditions, offering enhanced detection reliability and robustness in real-world deployment scenarios.

## 6. Conclusions

A GNSS spoofing detection method based on self-consistent verification of the receiver’s clock state is proposed in this paper. By analyzing the consistency between clock bias and clock drift, the method effectively captures anomalies introduced by spoofing in both pseudorange and Doppler measurements. Unlike conventional approaches that require complex bias extraction or auxiliary hardware, SCV-RCS is lightweight, easily integrable, and practical for real-world deployment.

The method was extensively validated through simulation, semi-hardware testing, and real-world experiments using the TEXBAT dataset. Tests across diverse spoofing scenarios demonstrated that SCV-RCS consistently outperforms existing methods in detection sensitivity, robustness, and sustained alerting capability. It achieves the fastest alarm latency (<2 s) in partial-channel and full-channel spoofing while maintaining a low false alarm rate and performs reliably across varying receiver motion states and attack configurations.

In summary, SCV-RCS provides a robust, real-time, and readily implementable defensive mechanism for GNSS receivers operating in adversarial signal environments. Future work will extend its applicability to more sophisticated spoofing models, such as multi-antenna attacks.

## Figures and Tables

**Table 1:** Simulation parameters for partial-channel spoofing scenario.

Parameters	Settings		
Date and Time	10 July 2015 00:00		
Utilized PRNs	2, 3, 6, 9, 10, 12, 17, 20, 23, 28		
Receiver Position	28.15625° N, 112.937° E, 100 m		
Spoofer Position	28.15641° N, 112.93606° E, 65 m		
Receiver Status	Case 1~case 8: Static Case 9~case 12: Uniform linear motion (v_x_ = 2 m/s)		
Carrier Frequency	1575.42 MHz (GPS L1 C/A)		
Duration/Spoofing Attack Moment	34 s/14 s		
Spoofing PRNs and Groups	G1: PRN 2, 3 G2: PRN 6, 9 G3: PRN 2, 3, 6, 9		
Replaying Delay	case 1: 0 m (G3) case 2: 100 m (G3) case 3: 150 m (G3) case 4: 300 m (G3)	case 5: 0 m (G1), 150 m (G2) case 6: 0 m (G1), 200 m (G2) case 7: 150 m (G1), 200 m (G2) case 8: 200 m (G1), 300 m (G2)	case 9: 0 m (G3) case 10: 50 m (G3) case 11: 100 m (G3) case 12: 200 m (G3)

**Table 2:** Simulation parameters for full-channel spoofing scenario.

Parameters	Settings		
Utilized PRNs	2, 3, 6, 9, 10, 12, 17, 20, 23, 28		
Receiver Status	Cases 1~4: Uniform circular motion (v = 6 m/s) Cases 5~8: Uniform linear motion (v_x_ = 3 m/s)		
Spoofing PRNs and Groups	G1: PRN 2, 3, 6, 9, 10 G2: PRN 12, 17, 20, 23, 28 G3: All		
Replaying Delay	Case 1: 0 m (G3) Case 2: 100 m (G3)	Case 3: 0 m (G1), 100 m (G2) Case 4: 100 m (G1), 200 m (G2)	Case 5: 0 m (G3) Case 6: 50 m (G3) Case 7: 100 m (G3) Case 8: 150 m (G3)

**Table 3:** Simulation parameters for semi-hardware simulation.

Parameters	Settings	
Date and Time	11 February 2025 08:00	
Utilized PRNs	PRN 4, 7, 8, 11, 17, 20, 27, 28, 32	
Receiver Position	28.15625° N, 112.937° E, 100 m	
Spoofer Position	28.15633° N, 112.93653° E, 82.82 m	
Receiver Status	Case 1~case 4: Uniform circular motion (v = 6 m/s) Case 5~case 8: Uniform linear motion (v_x_ = 2 m/s)	
Carrier Frequency	1575.42 MHz (GPS L1 C/A)	
Duration/Spoofing Attack Moment	60 s/37 s	
Spoofing PRNs and Groups	Group 1: All (full channel) Group 2: PRN 4, 7, 8, 11 (partial channel)	
Replaying Delay	Case 1: 0 m (Group 1) Case 2: 50 m (Group 1) Case 3: 100 m (Group 1) Case 4: 150 m (Group 1)	Case 5: 0 m (Group 2) Case 6: 50 m (Group 2) Case 7: 100 m (Group 2) Case 8: 150 m (Group 2)

## Data Availability

TEXBAT is a public dataset, generated by team Humphreys, where the TEXBAT can be downloaded from the website https://rnl-data.ae.utexas.edu/datastore/texbat/ (accessed on 12 December 2025).
